# Development and validation of a tool to assess core competencies of public health professionals in low-income settings: findings from Uttar Pradesh, India

**DOI:** 10.1186/s12960-025-00994-5

**Published:** 2025-06-23

**Authors:** Sudip Bhandari, Sara Bennett, David H. Peters

**Affiliations:** 1 Johns Hopkins University, Bloomberg School of Public Health, Baltimore, USA; 2 York University, Faculty of Health, Toronto, Canada

**Keywords:** Public health competencies, Index, Psychometric evaluation, Factor analysis, Human resource development, India

## Abstract

**Background:**

Many low- and middle-income countries (LMICs) lack instruments to measure gaps in the public health competency of health professionals. The objective of this study is to develop a validated and reliable Core Public Health Competency (COPHEC) index by assessing the knowledge, skills, abilities, and attitudes of senior and mid-level public health professionals with supervisory and management responsibilities in Uttar Pradesh (UP), India.

**Methods:**

Using the Core Competency framework that was developed in UP, we generated a draft COPHEC tool with 37 items, measured on a four-point Likert scale. We administered the tool to a total of 166 public health professionals that included two samples—84 senior and 82 mid-level public health professionals. To extract factors and assign factor scores to the instrument, we performed an exploratory factor analysis (EFA) using principal component analysis (PCA). Content and face validities were assessed by examining the steps used for the construction of the draft tool. Construct validity was measured by assessing the average factor loading of the items onto the component extracted from EFA. Internal consistency was used as a measure of reliability.

**Results:**

The final COPHEC index had 37 items loaded on one factor in the sample. Content and face validities were assured through a participatory process with relevant stakeholders who identified the initial set of items as part of a Core Competency framework. Construct validity of the COPHEC scale was confirmed by the high average factor loading of components ranging from 0.58 to 0.81. The final index showed adequate reliability with Cronbach’s alpha (*α*) = 0.97.

**Conclusions:**

The COPHEC index is a valid and reliable measure of core competencies in public health in UP. We recommend that governments adapt the index in LMICs to conduct assessments of health workers to identify training needs, evaluate the effectiveness of training programs through participants’ competency acquisition pre- and post-training, and inform workforce development efforts in recruitment and performance management.

## Background

Achieving Universal Health Coverage (UHC) requires that health professionals with public health responsibilities have adequate knowledge, skills, abilities, and attitudes to deliver essential public health services [1–6]. Core competencies are the critical knowledge, skills, abilities, and attitudes that the health workforce should possess to effectively deliver essential public health functions, such as epidemiological surveillance, situation assessments, and health promotion [7, 8]. They draw on multiple public health disciplines and are not specific to a single program or topic. A recent World Health Organization report on the public health workforce highlights the importance of competencies in responding to ongoing and future challenges, such as pandemics, climate change, rising noncommunicable disease burden, and antimicrobial resistance [9]. However, many low-resource settings, including the state of Uttar Pradesh (UP) in India, struggle to ensure that the health workforce has the appropriate competencies needed to effectively perform public health functions [10, 11].

UP is the most populous state in India, with almost 230 million people [12]. As with many states in the country, UP continues to face several health workforce challenges. There are currently an estimated 22.1 health workers per 10,000 population compared to the WHO proposed Sustainable Development Goal index threshold of 44.5 health workers per 10,000 [13, 14]. There are no requirements and few opportunities for health workers to receive public health training, making it challenging to deliver essential public health functions (EPHFs) for population health or professionally manage health services. In addition, there are discrepancies between professional competencies and population health priorities, an unsuitable mix of competencies among the health workforce, and a maldistribution of professionals across geographical areas—specifically rural and urban regions [10, 15].

Identifying a set of core competencies for public health professionals is an important step in addressing these challenges. To that end, there has been much discussion about developing competencies in the public sector and academia in India. For instance, the Department of Personnel and Training of the Government of India, in collaboration with the United Nations Development Program (UNDP), started a competency-based system of strategic human resource management for the Indian Civil Service in 2011 [16]. The outcome of this effort was a “Competency Dictionary” that identified 25 core competencies across various roles and positions of civil service employees [17]. Related to this effort are the National Training Policy of 2012 and the Framework of Roles, Activities, and Competencies (FRAC) document of 2020 which highlighted the importance of competencies, asserting that career progression and recruitment in public health jobs should be based on the individual competencies required for those posts [18, 19]. Similarly, the Indian national health policy of 2017 discusses the role of competency-based courses as a way to develop the cadre of mid-level primary care providers [20]. The Ministry of Health and Family Welfare (MoHFW) and other researchers have developed frameworks to define core public health competencies for Masters of Public Health (MPH) programs in India [21, 22], but to-date efforts to identify core competencies for public health professionals in India and assess competency gaps are limited.

Our recent study built on prior critical efforts to identify the requisite core competencies for health professionals in mid-level supervisory and program management roles in UP [23]. However, there is still a need for validated and reliable tools to measure competencies among public health professionals. Most of the available metrics related to human resources for health in low- and middle-income countries (LMICs) focus on the availability, production, and distribution of health workers [24–26]. The limited number of tools used in LMIC settings that do focus on competencies are directed mostly at clinical health workers, such as medical doctors and nurses [27, 28]. Even these instruments tend to focus on selective areas of clinical practice. To address these gaps, this paper develops a validated and reliable tool that can be used to quantitatively assess core competencies of public health professionals in the state of Uttar Pradesh in India.

## Methods

The selection of respondents for the study was guided by our interests and the need to learn about competency gaps among public health professionals at the state, district, and block levels—the three administrative divisions in UP (and India). Our sample of senior professionals consisted of all senior health professionals at the state level, and it provides a comprehensive picture of public health competency at that level. Our sample of Medical Officers provides a good indication of the public health competency gaps in recently hired mid-level professionals at the district and block levels.

**Fig. 1:**
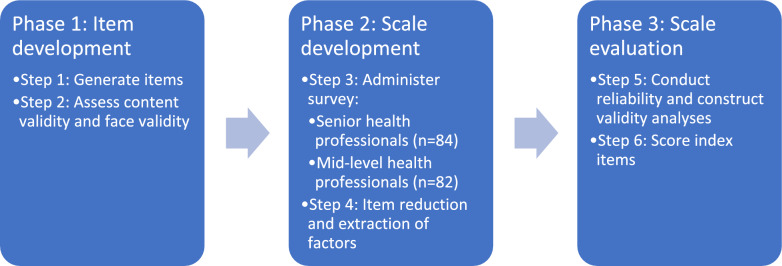
Three-phase COPHEC tool development process

Procedures for this study were broadly informed by the phases and steps outlined by Boateng and colleagues for developing and validating scales in health research [29]. Each phase and associated steps in this study are described below and in Fig. 1.

### Phase 1: Item development

#### Step 1: Generate items

In this step, we first specified the purpose of the construct we were seeking to develop, which was to create a composite measure of cumulative public health knowledge, skill, ability, and attitude of health professionals with public health responsibilities. Second, through the review of relevant literature, we confirmed that there are no existing instruments that measure public health competencies in the UP setting or India at large.

Third, we utilized the framework of Core Competencies for Public Health Professionals in Uttar Pradesh for item generation [23]. This framework, generated through a structured participatory process of Indian experts identifies 48 competency statements organized across eight public health domains, such as public health sciences and financial management and budgeting. We consolidated similar statements into an initial tool of 37 items, merging homogenous items and retaining heterogeneous ones to minimize participant burden and survey fatigue, aligning with research on survey length [30].

#### Step 2: Assess content validity and face validity

Establishing content validity for this tool involved evaluating the degree to which the initial set of 37 items represented the “universe” of the construct of core public health competency. Content validity in this context meant the degree to which the initial 37 items covered all aspects of the core public health competency construct they aimed to measure [31].

Maintaining face validity in this study meant evaluating the acceptability of the core competency assessment instrument to the users and respondents in the UP context. Face validity in this context is the degree to which the competency assessment instrument appears to measure what it’s supposed to measure in the eyes of topic experts and those who would use the measure [31].

The following steps, which were taken as a part of the multi-step Delphi process to develop the framework, ensured that the framework was valid in the UP setting [23]. A narrative review synthesized global competency frameworks into a preliminary list relevant to UP. Rapid semi-structured qualitative interviews with Indian experts refined the list. This updated preliminary list of competencies became the starting point for a day-long workshop, which eventually generated a competency framework based on consensus. This panel of Indian public health experts and government officials had the knowledge and expertise to develop a framework that was relevant to and representative of the targeted construct. The process of discussion, debate, amendment, and eventually finalization in this group was particularly relevant in ensuring the content and face validity of the eventual competency items. These experts and officials asserted that the identified competencies covered the necessary public health domains in the UP context (establishing content validity) and, on the face of it, were appropriate for mid-level health officials (establishing face validity).

These validation steps, in turn, ensured the content validity and face validity of the initial 37-item tool as well. The competency statements were consolidated and refined to create the initial items in the COPHEC tool. The details of these and other steps of the competency identification process, which formed Phase 1 of this research, are presented elsewhere [23].

The refinement of the initial 37-item tool with the help of local experts through translation and back translation also contributed to the face validity of the instrument. A team from an Indian language services company conducted the initial translation of the tool items into Hindi. Then a back-translation of the instrument from Hindi to English was conducted through another Indian translator fluent in both English and Hindi. This translator had not seen the questionnaire before and did not have access to the first English draft. Once the translation and back-translation processes were complete, the first author and members of the research team reviewed these documents over the course of several meetings. In these meetings, the team discussed the intent of each item, the literal translation, commonly understood the meaning of the item, and the appropriateness of the word choice and phrasing in Hindi as well as English. The final version of the tool is shown in Appendix 1.

### Phase 2: Scale development

#### Step 3: Administer the survey

The 37-item tool was administered among health professionals in UP in two samples using pen-and-paper data collection. The first sample was a census of senior health professionals who worked in the Directorate of Medical and Health and the Directorate of Health and Family Welfare in Lucknow, the capital district of UP (*N* = 84). The survey for this sample was conducted from September to November 2019. The second sample was a convenience sample of recently hired mid-level health professionals who worked mainly in Primary Health Centers (PHCs) across the state (*N* = 82). For this sample, we approached those who were present for pre-service training at the State Institute of Health and Family Welfare (SIHFW), the government of UP’s training institute. The participants belonged to three consecutive training batches. The surveys for the first, second, and third batches were conducted in September 2019, November 2019, and February 2020, respectively.

#### Step 4: Item reduction and extraction of factors

Data analysis was performed using STATA 14.0 statistical package [32]. Data analysis followed steps for new scale construction identified by DeVellis [33]. Exploratory Factor Analysis (EFA) was utilized to identify a parsimonious list of factors that describe the core public health competencies (COPHEC) and generate factor scores that can then be used for subsequent analyses. EFA is a useful technique for studying competency, given the latent nature of the construct. Many competencies listed in the tool are not directly observed, and they can only be inferred through mathematical models. The method has been used for developing competency frameworks, determining indicators for competency assessment, and validating competency scales [34–37].

**Fig. 2:**
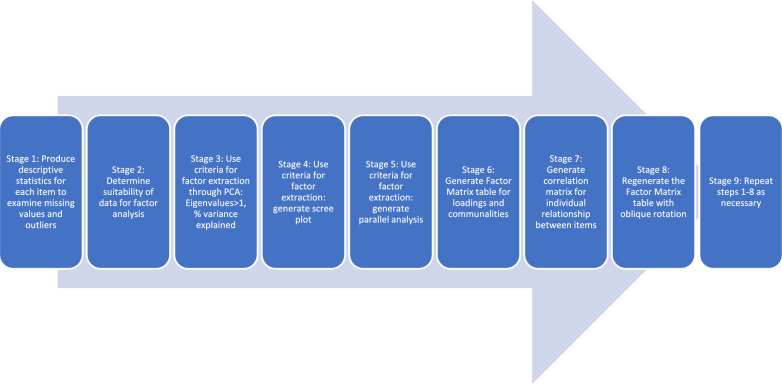
Factor analysis steps used in the analysis

To conduct an EFA, we followed nine stages (Fig. 2). First, we assessed each item descriptively for missing values and outliers. Second, we tested data suitability for factor analysis using the test of “determinant”, Kaiser–Meyer–Olkin (KMO) test of sampling adequacy, and Bartlett’s tests [38, 39]. We required the “determinant” test to be unequal to 0, KMO value of ≥ 0.6, and a significant Bartlett’s test (*p* < 0.05). Third, Principal Component Analysis (PCA) provided Total Variance Explained, selecting components with Eigenvalues > 1, and variables cumulatively explaining > 50% variance [40]. Fourth, scree plots identified factors before the leveling off (the “elbow”) [41]. Fifth, parallel analysis compared the eigenvalues generated from the data matrix to the eigenvalues generated from a Monte-Carlo simulated matrix created from a random set of equivalent size [42, 43]. We then compared the results of these four methods—Eigenvalues greater than 1, variance explained, scree plot, and parallel analysis to decide on the number of factors to retain.

Sixth, we created a Factor Matrix table to show the loadings of each item on each factor and the communalities of the item [44]. The value of 0.5 was used as the criterion for minimum factor loading and 0.3 as the minimum communality for that item to be retained [45]. Seventh, we created a correlation matrix to evaluate inter-item correlation and drop items with high correlations (greater than 0.8). Items with cross-loadings or that appear not to load uniquely on individual factors were deleted. Eighth, once we made the initial decision on the number of factors and items to retain based on the above steps, we regenerated the Factor Matrix table with oblique rotation to improve the interpretation of the factor. We chose oblique rotation—instead of orthogonal rotation—because we thought that the underlying latent variables might be somewhat correlated with one another. Finally, we repeated steps 6, 7, and 8 as necessary, considering different items and the number of factors. The factors were then used to generate factor scores.

### Phase 3: Scale evaluation

#### Step 5: Conduct reliability and construct validity analyses

For reliability, Cronbach’s alpha was used to assess the internal consistency of the final scale items belonging to the same factor [46]. An alpha coefficient of 0.70 was used as the criterion for the acceptable threshold for this reliability.

Construct validity refers to how well the items on a questionnaire represent the underlying conceptual structure [47, 48]. By examining the factor loadings in the rotated matrix generated from the EFA, we assessed how different items align into conceptual constructs that describe core public health competencies.

#### Step 6: Score index items

Finalized items from the above steps were used to generate one factor and a set of factor scores. Factor scores were calculated using the post-estimation procedure. These are composite variables that provide information about an individual’s placement on the factor identified from the EFA [49]. After the tool was developed, the results were analyzed separately for senior and mid-level health professionals.

## Results

### Characteristics of the health professionals surveyed

Table 1 presents the demographic characteristics of the respondents disaggregated by seniority level. In terms of public health training received, just over 80% of the senior professionals reported having received some in-service public health training. All mid-level professionals on the other hand reported receiving public health training, which is accurate given that all the respondents had recently attended the medical officer foundational training.

**Table 1:** Demographic characteristics of study participants disaggregated by seniority level

SN	Participants characteristics	Senior professionals (*n* = 84)	Mid-level professionals (n = 82)	Total (N = 166)
1	Length of service in the UP’s health system (mean years, Std. Dev.)	27.59 (7.03)	2.48 (1.82)	15.19 (13.61)
2	Length of service in the current position (mean years, Std. Dev.)	3.34 (2.62)	2.32 (1.77)	2.84 (2.29)

		*n*	%	*n*	%	*n*	%
3	Sex						
	Male	66	79%	55	67%	121	73%
	Female	18	21%	27	33%	45	27%
4	Current title (by seniority)^1^					All	
4a	*Senior professionals*^*2*^						
	Director	11	13%	0	0	11	7%
	Additional Director (AD)	13	15%	0	0	13	8%
	Joint Director (JD)	56	67%	0	0	56	34%
	Other	4	5%	0	0	4	2%
4b	*Mid-level professionals*^*3*^						
	Medical officer in charge (MOIC)	0	0	5	6%	5	3%
	Medical officer (MO)	0	0	77	94%	77	46%
5	Some public health training received						
	Yes	68	81%	82	100%	150	90%
	No	16	19%	0	0	16	10%
6	Highest education level						
	Bachelors	61	73%	78	95%	139	84%
	Master’s	6	7%	2	2%	8	5%
	Doctorate	17	20%	2	2%	19	11%

^1^The titles of Director, AD, and JD are only applicable to senior health professionals, so these options were not provided to the sample of mid-level health professionals. Similarly, the titles of MO and MOIC are only applicable to mid-level health professionals, so these options were not provided to the sample of senior health professionals

^2^In the directorate of medical and health and directorate of health and family welfare, directors are ranked higher than Additional Directors (ADs), who are ranked higher than Joint Directors (JDs). Each of the 12 departments (e.g., training, paramedical, nursing) in the directorate of medical and health, and the two departments (family welfare, and maternal and child welfare) in the directorate of health and family welfare typically have one sanctioned director, between one to four ADs, and between one to six JDs

^3^A Medical Officer-In-Charge (MOIC) is usually the head of a Community Health Center (CHC) in a block, while an MO leads the Primary Health Center (PHC)

### Factor analysis

Missing data analysis showed that data were Missing Completely at Random (MCAR) (see Appendices 2 and 3). We used Kaiser–Meyer–Olkin (KMO) and Bartlett’s test of sphericity to confirm the suitability of data for factor analysis. The KMO value for the COPHEC index was 0.93, indicating that underlying factors might explain the proportion of variance in the variables. Bartlett’s test of sphericity was *χ*^2^ (666) = 4303, *p* < 0.0001, indicating that the correlation between items was sufficiently large to run a PCA. Both tests indicated that the use of factor analysis was appropriate. The correlation matrix showed there were no items with a correlation greater than 0.8, so no items were deleted based on this criterion (see Appendix 4).

The PCA suggested one component explaining 53% of the variation in the initial 37-item scale solution for the sample (see Appendix 5). The scree plot suggested the retention of one component (see Appendix 6). The parallel analysis confirmed the retention of one component (see Appendices 7 and 8). This one factor—which we named core public health competency—was used to generate the COPHEC index.

During the extraction process, all items met the minimum threshold of factor loadings and communalities, so no items were deleted (see Table 2). Table 2 includes the pattern matrix, showing the correlation between each of the final items and the component extracted after the iterative process.

**Table 2:** Pattern matrix and factor scoring coefficients of the final COPHEC index

Item #	Item	Factor loading	Uniqueness	Factor scoring coefficients
CA.01	Describe key concepts in public health (e.g., the health status of populations, the determinants of health and illness, strategies for health promotion, the relationship between health and poverty, inequities in health and various forms of disadvantages, disease and injury prevention and health protection, as well as the factors that influence the delivery and use of health services.)	0.604	0.635	0.022
CA.02	Apply the public health tools, techniques, and sciences (e.g., behavioral and social sciences, biostatistics, economics, epidemiology, environmental public health, demography) to practice (e.g., community needs assessment)	0.652	0.575	0.027
CA.03	Use data, evidence, and research to inform health policies, programs, and organizational performance	0.674	0.546	0.029
CA.04	Identify relevant and appropriate sources of information, including community resources	0.692	0.521	0.031
CA.05	Collect, store, retrieve, and use accurate and appropriate data on public health issues	0.699	0.512	0.032
CA.06	Analyze information to determine appropriate implications, uses, gaps, and limitations	0.687	0.529	0.031
CA.07	Assess the accuracy and importance of data for public health decision making	0.680	0.537	0.030
CA.08	Describe selected policy and program options to address a specific public health issue	0.716	0.488	0.035
CA.09	Describe the implications of each policy and program option, especially as they apply to the determinants of health and recommend or decide on a course of action	0.677	0.542	0.030
CA.10	Develop a plan to implement a course of action taking into account relevant evidence, emergency planning procedures, regulations and policies, and legislation (e.g., government order)	0.754	0.431	0.041
CA.11	Implement a policy, program, or effective practice guidelines (e.g., immunization guidelines, screening programs for illnesses) to address a specific public health issue	0.756	0.428	0.042
CA.12	Monitor and evaluate an action, policy, or program	0.710	0.497	0.034
CA.13	Demonstrate the ability to fulfill functional roles in response to a public health emergency	0.766	0.414	0.044
CA.14	Establish teams for the purpose of achieving program and organizational goals (e.g., considering the value of different disciplines, sectors, skills, experiences, and perspectives; determining scope of work and timeline)	0.805	0.352	0.054
CA.15	Motivate and supervise personnel for the purpose of achieving program and organizational goals (e.g., participating in teams, encouraging sharing of ideas, respecting different points of view)	0.702	0.508	0.033
CA.16	Support learning within an organization including on the job training to advance public health goals	0.690	0.525	0.031
CA.17	Manage time appropriately	0.600	0.640	0.022
CA.18	Justify programs for inclusion in budgets, develop and defend budgets	0.630	0.603	0.025
CA.19	Prepare proposals for funding (e.g., foundations, government agencies, corporations)	0.648	0.580	0.026
CA.20	Make use of financial analysis and accounting techniques in making decisions about policies, programs, and services	0.578	0.666	0.021
CA.21	Manage programs within current and projected budgets and staffing levels (e.g., sustaining a program when funding and staff are cut, recruiting and retaining staff)	0.636	0.596	0.025
CA.22	Identify and collaborate with partners in addressing public health issues	0.773	0.403	0.045
CA.23	Use skills such as team building, negotiation, conflict management, group facilitation, and mediation between differing interests to build partnerships	0.809	0.345	0.055
CA.24	Mobilize communities using appropriate media, community resources, and social marketing techniques	0.737	0.456	0.038
CA.25	Recognize how the determinants of health (biological, social, cultural, economic and physical) influence the health and well-being of specific population groups	0.729	0.469	0.037
CA.26	Address population diversity when planning, implementing, adapting, and evaluating public health programs and policies	0.756	0.429	0.042
CA.27	Apply culturally relevant and appropriate approaches with people from diverse castes, religions, socioeconomic and educational backgrounds, and persons of all ages, genders, health status, sexual orientations and abilities	0.735	0.459	0.038
CA.28	Listen, engage, and communicate effectively (e.g., by leveraging technology) with individuals, families, groups, communities, and colleagues including supervisors and team members	0.759	0.425	0.042
CA.29	Interpret information for professional, nonprofessional and community audiences	0.801	0.359	0.053
CA.30	Advocate and network for healthy public policies and services that promote and protect the health and well-being of individuals and communities	0.726	0.474	0.036
CA.31	Describe the mission and priorities of the public health organization, where one works, and apply them in practice	0.757	0.426	0.042
CA.32	Contribute to developing key values and a shared vision in planning and implementing public health programs and policies in the community	0.776	0.397	0.046
CA.33	Utilize public health ethics to manage self, others, information, and resources	0.796	0.366	0.051
CA.34	Contribute to maintaining organizational performance standards	0.749	0.440	0.040
CA.35	Build community capacity by sharing knowledge, tools, expertise, and experience	0.753	0.433	0.041
CA.36	Identify a need for change, manage change and processes	0.734	0.462	0.038
CA.37	Maintain organizational justice, equality, and fairness in dealing with subordinates	0.682	0.535	0.030

### Factor scores

The factor score coefficients for each item in the final 37-item COPHEC index are listed in Table 2. The scores, generated after rescaling the standardized factor score to between 0 to 100, showed an overall mean competency score of 58.61 (SD = 19.36). Mid-level health professionals averaged 63.4 (SD = 15.74) in their competencies, while senior-level professionals averaged 54.8 (SD = 21.7).

### Reliability and validity assessments

#### Reliability

Analysis of inter-item correlation showed high internal consistency. We found Cronbach’s alpha to be 0.97 for the full 37-item index.

#### Content validity and face validity

Content validity and face validity of the COPHEC index were assured primarily through the methodological rigor and participatory nature of the process to design the Core Competencies framework. In addition, the face validity of the instrument was strengthened through the refinement of the tool through the translation and back-translation process.

#### Construct validity

Construct validity of the COPHEC index was confirmed by the high average factor loading of components ranging from 0.58 to 0.80. Ideally, we would examine the components correlation matrix for the rotated final components as another measure of construct validity. However, we were unable to do so, because we were left with only one component in the final tool.

### Results of the tool’s implementation

The competency assessments using the COPHEC tool yielded several noteworthy findings. The majority of the respondents self-rated themselves as either aware (competency score of 2) or knowledgeable (score of 3) on a scale of 1 to 4. Across all the competency statements, a higher percentage of mid-level professionals rated themselves as knowledgeable (score of 3) or proficient (score of 4) compared to senior-level professionals.

A significant difference was observed in the competency related to applying public health tools, techniques, and sciences to practice (item #CA.02). Sixty-eight percent of mid-level professionals considered themselves either knowledgeable or proficient (score of 3 or 4 out of 4) in this competency, compared to only 35% of senior professionals. In contrast, the smallest difference (2%) between the two groups was found in the competency for managing time appropriately (item #CA.17), with 80% of mid-level professionals and 78% of the senior professionals rating themselves as knowledgeable or proficient (score of 3 or 4) in this competency. In addition, in the competency about demonstrating the ability to fulfill functional roles in response to a public health emergency (item #CA.13), 22% of the mid-level professionals and 33% of the senior professionals rated themselves as either having no competency or only aware (score of 1 or 2).

Other key gaps were also found. Among mid-level health professionals, 81% rated themselves to be knowledgeable or proficient (score of 3 or 4) in utilizing public health ethics to manage self, others, information, and resources (item #CA.33). On the other hand, the competencies where mid-level health staff seemed particularly poorly prepared were financial ones where 42% said they had no competency or were only aware (score of 1 or 2) about preparing proposals for funding (item #CA.19) and 48% reporting similar levels of competency in interpreting financial data (item #CA.20).

Senior health professionals demonstrated strengths in maintaining organizational justice, equality, and fairness in dealing with subordinates (item #CA.37), with 75% rating themselves as knowledgeable or proficient (score of 3 or 4). However, 61% of senior professionals reported no competency or only awareness (score of 1 or 2) in preparing proposals for funding (item #CA.19), 66% in using financial analyses (item #CA.20), and 62% in managing programs with budgets (item #CA.21). For further details, please refer to Appendix 9. Self-assessed competency levels, stratified by mid-level versus senior professionals for the COPHEC tool.

## Discussion

This study presents a novel tool to assess self-reported core public health competencies in UP and broadly in India. We envision the COPHEC index to apply and be relevant to both mid-level and senior officials. While the required competencies might be similar between the two groups, varying levels of proficiency in different competencies may be required depending on the nature of job responsibilities.

The results from the COPHEC tool reveal critical insights into the competency levels of healthcare professionals. Notably, there are distinct differences in self-assessed competencies between mid-level and senior professionals, particularly in areas, such as public health ethics, managing finances, and proposal preparation. These disparities highlight potential areas for targeted training and development, especially in financial resource mobilization and the practical application of public health tools and techniques. In addition, the findings suggest a higher self-assessment of competency levels among mid-level professionals compared to senior professionals in various domains, indicating possible gaps in ongoing professional development or differences in job responsibilities and exposure to practical applications. Mid-level professionals could also be overestimating their competencies, a phenomenon called the Dunning–Kruger effect, a cognitive bias in which respondents assess their ability as higher than it is [50]. Previous research has demonstrated that less competent individuals tend to misjudge their competencies, because they are usually not in a position to recognize their cognitive shortcomings. In other words, they do not know what they do not know. The scope of their ignorance is often invisible to them, because they lack the expertise and knowledge to recognize how deficient their competence is [51].

These findings underscore the necessity for tailored professional development programs that address the specific needs of different professional levels. For example, while mid-level professionals might benefit from advanced training in leadership and resource management, senior professionals may require refresher courses to update their skills in practical public health tools and techniques. The consistent disparities observed between the two groups also suggest that a one-size-fits-all approach to training will not be effective. Instead, differentiated training programs that consider the varying competencies and job responsibilities could enhance the overall effectiveness of public health interventions.

The findings of this study have important implications for policy and planning in public health education and workforce development in Uttar Pradesh. Insights from the COPHEC tool can guide targeted educational curricula and training programs to address competency gaps in areas, such as public health ethics, financial management, and emergency response. Emphasizing ongoing professional development through structured training will ensure health professionals can effectively meet evolving public health challenges. Integrating competency assessments into policy frameworks can strengthen Uttar Pradesh’s public health workforce and enhance healthcare delivery for its population.

The EFA in this study identified one factor that explained the majority of the variance in our data. This finding is different than what we saw in the scientific literature from high-income countries, where EFA analysis of public health competencies has yielded up to ten domains [52, 53]. Our findings in this study are also different than our previous study which had eight domains. This suggests that further research in UP may be necessary to confirm the factor structure.

Future research on the COPHEC tool holds promising avenues for advancing our understanding of public health competency assessment. We propose three distinct areas that warrant exploration and refinement. The first area centers on the further calibration of the COPHEC tool, particularly through confirmatory factor analysis. Extending this analysis to diverse samples within UP or similar resource-poor settings will validate the factor structure of the COPHEC index. Moreover, exploratory factor analyses conducted in analogous resource-poor contexts across India and other LMICs can yield insights to refine the instrument.

A second area of research is the comparison of self-assessment with other assessment methods. Investigating how self-assessment aligns with objective evaluation techniques—such as standardized exam questions or vignette tests—will enrich our understanding of the COPHEC index’s validity. We plan to examine this further in future analyses of participants of this study.

The third area of future research includes exploring the practical applications of competency measurement, including the integration of competency assessments into licensure and accreditation requirements for public health professionals, akin to practices in aviation and clinical medicine. This exploration should assess the capacity of government agencies in UP and similar settings to implement such systems, potentially using technology like digital badges to streamline processes. Longitudinal and repeated cross-sectional designs should be employed to monitor competency change over time to inform the sustainability of training interventions and factors that contribute to such sustenance, and to guide programmatic decisions such as the frequency of in-service training programs.

A strength of this study is that it utilized many recommendations for the development of psychometric scales, which helped in producing a tool with good validity and reliability. However, several limitations should be noted. The first relates to sampling. The study included a convenience sample of mid-level health professionals present in the training program offered by SIHFW. We are unable to determine the extent of bias in our sample, because we do not know how these trainees were selected at an individual level. Therefore, the results from this study should be interpreted cautiously, as findings may not be generalizable to all the senior and mid-level health professionals.

The second limitation relates to self-assessment. Studies in fields such as clinical medicine have demonstrated that self-assessments tend to be less reliable predictors of actual competencies compared to objective or third-party assessments of competencies [54].

The third limitation is the subject-to-item ratio related to the sample size for EFA. The ratio for this study was just over four, lower than the ratio of five to ten suggested by some researchers [55–58]. However, studies show that these recommendations are inconsistent, and there is little empirical evidence to support them, so they are gradually being abandoned [59–62]. There is also research that shows that even with a smaller sample size, the findings of an EFA can be valid under conditions, where there are high factor loadings, few factors, and high communalities, which are characteristic of the results in this study [61–65].

## Conclusion

Despite the recognition that improving health workers’ competencies is important to achieve UHC, most human resources for health indicators only measure the availability and distribution of health workers. Indicators that do focus on competencies relate mostly to the clinical workforce. While there are very few studies that have measured public health competencies, they come from HICs and there are no reliable and validated tools for such measurement in low-resource settings like UP. The 37-item COPHEC index helps to fill that gap—it was found to be a valid and reliable measure of core public health competency among health professionals with management and supervision roles in UP.

The tool presented here would be most useful if ministries of health initiate the assessment to generate productive discussions around current capacities to meet public health needs, including response to public health emergencies. Specifically, they can use this instrument to inform training programs based on competency assessment gaps; evaluate training effectiveness by measuring competency acquisition before and after training; assess the level of competencies among potential recruits to make hiring decisions; improve performance management including the promotion of adequately competent professionals; and incentivize in-service training programs to improve certain competencies among health workers.

## Data Availability

The data used and analyzed during the study are available from the corresponding author on reasonable request.

## Appendices

### Appendix 1 Final self-assessment competency assessment tool

**Table:**

For each statement, think about the level at which you are currently able to perform the skill. Then rate your level of proficiency on each statement by selecting a number between 1 and 4: 1 = None; I am unaware or have very little knowledge of the skill 2 = Aware; I have heard of, but have limited knowledge or ability to apply the skill 3 = Knowledgeable; I am comfortable with my knowledge or ability to apply the skill 4 = Proficient; I am very comfortable, am an expert, or could teach this skill to others Note: The statements should be interpreted as broadly as possible to apply to your position and main setting of your work. In the example below, you would select number “4” for “Proficient” if you think you are excelling at this skill or select “1” for “None” if you feel you need a great deal of improvement	

Item number	Survey item
CA.01	Describe key concepts in public health (e.g., the health status of populations, the determinants of health and illness, strategies for health promotion, relationship between health and poverty, inequities in health and various forms of disadvantages, disease and injury prevention and health protection, as well as the factors that influence the delivery and use of health services.)
CA.02	Apply the public health tools, techniques, and sciences (e.g., behavioral and social sciences, biostatistics, economics, epidemiology, environmental public health, demography) to practice (e.g., community needs assessment)
CA.03	Use data, evidence, and research to inform health policies, programs, and organizational performance
CA.04	Identify relevant and appropriate sources of information, including community resources
CA.05	Collect, store, retrieve and use accurate and appropriate data on public health issues
CA.06	Analyze information to determine appropriate implications, uses, gaps, and limitations
CA.07	Assess the accuracy and importance of data for public health decision making
CA.08	Describe selected policy and program options to address a specific public health issue
CA.09	Describe the implications of each policy and program option, especially as they apply to the determinants of health and recommend or decide on a course of action
CA.10	Develop a plan to implement a course of action taking into account relevant evidence, emergency planning procedures, regulations and policies, and legislation (e.g., government order)
CA.11	Implement a policy, program, or effective practice guidelines (e.g., immunization guidelines, screening programs for illnesses) to address a specific public health issue
CA.12	Monitor and evaluate an action, policy, or program
CA.13	Demonstrate the ability to fulfill functional roles in response to a public health emergency
CA.14	Establish teams for the purpose of achieving program and organizational goals (e.g., considering the value of different disciplines, sectors, skills, experiences, and perspectives; determining scope of work and timeline)
CA.15	Motivate and supervise personnel for the purpose of achieving program and organizational goals (e.g., participating in teams, encouraging sharing of ideas, respecting different points of view)
CA.16	Support learning within an organization including on the job training to advance public health goals
CA.17	Manage time appropriately
CA.18	Justify programs for inclusion in budgets, develop and defend budgets
CA.19	Prepare proposals for funding (e.g., foundations, government agencies, corporations)
CA.20	Make use of financial analysis and accounting techniques in making decisions about policies, programs, and services
CA.21	Manage programs within current and projected budgets and staffing levels (e.g., sustaining a program when funding and staff are cut, recruiting and retaining staff)
CA.22	Identify and collaborate with partners in addressing public health issues
CA.23	Use skills such as team building, negotiation, conflict management, group facilitation, and mediation between differing interests to build partnerships
CA.24	Mobilize communities using appropriate media, community resources, and social marketing techniques
CA.25	Recognize how the determinants of health (biological, social, cultural, economic and physical) influence the health and well-being of specific population groups
CA.26	Address population diversity when planning, implementing, adapting, and evaluating public health programs and policies
CA.27	Apply culturally relevant and appropriate approaches with people from diverse castes, religions, socioeconomic and educational backgrounds, and persons of all ages, genders, health status, sexual orientations and abilities
CA.28	Listen, engage, and communicate effectively (e.g., by leveraging technology) with individuals, families, groups, communities, and colleagues including supervisors and team members
CA.29	Interpret information for professional, nonprofessional and community audiences
CA.30	Advocate and network for healthy public policies and services that promote and protect the health and well-being of individuals and communities
CA.31	Describe the mission and priorities of the public health organization where one works, and apply them in practice
CA.32	Contribute to developing key values and a shared vision in planning and implementing public health programs and policies in the community
CA.33	Utilize public health ethics to manage self, others, information, and resources
CA.34	Contribute to maintaining organizational performance standards
CA.35	Build community capacity by sharing knowledge, tools, expertise, and experience
CA.36	Identify a need for change, manage change and processes
CA.37	Maintain organizational justice, equality, and fairness in dealing with subordinates

### Appendix 2 Number and percentage of missing values for each item

**Table:**

Item number*	Missing	Total	Percent Missing	Item number	Missing	Total	Percent Missing
CA1	4	166	2.41	CA23	4	166	2.41
CA2	4	166	2.41	CA24	4	166	2.41
CA3	5	166	3.01	CA25	4	166	2.41
CA4	8	166	4.82	CA26	6	166	3.61
CA5	6	166	3.61	CA27	7	166	4.22
CA6	5	166	3.01	CA28	5	166	3.01
CA7	5	166	3.01	CA29	8	166	4.82
CA8	3	166	1.81	CA30	4	166	2.41
CA9	4	166	2.41	CA31	5	166	3.01
CA10	4	166	2.41	CA32	4	166	2.41
CA11	4	166	2.41	CA33	3	166	1.81
CA12	4	166	2.41	CA34	8	166	4.82
CA13	8	166	4.82	CA35	3	166	1.81
CA14	4	166	2.41	CA36	4	166	2.41
CA15	2	166	1.2	CA37	3	166	1.81

*Item corresponds to the item number Appendix 1

### Appendix 4 Correlation matrix

**Table:**

Items	CA1	CA2	CA3	CA4	CA5	CA6	CA7	CA8	CA9	CA10	CA11	CA12	CA13	CA14	CA15	CA16	CA17	CA18	CA19
CA1	1.00																		
CA2	0.63	1.00																	
CA3	0.61	0.66	1.00																
CA4	0.56	0.54	0.58	1.00															
CA5	0.56	0.52	0.66	0.70	1.00														
CA6	0.57	0.44	0.57	0.55	0.73	1.00													
CA7	0.54	0.54	0.63	0.54	0.71	0.76	1.00												
CA8	0.52	0.55	0.74	0.63	0.68	0.61	0.66	1.00											
CA9	0.60	0.48	0.54	0.58	0.60	0.58	0.57	0.56	1.00										
CA10	0.57	0.50	0.58	0.60	0.65	0.58	0.57	0.56	0.70	1.00									
CA11	0.54	0.49	0.56	0.47	0.49	0.52	0.48	0.54	0.62	0.62	1.00								
CA12	0.45	0.38	0.52	0.50	0.53	0.55	0.50	0.57	0.56	0.58	0.73	1.00							
CA13	0.44	0.41	0.50	0.53	0.52	0.49	0.50	0.60	0.48	0.53	0.64	0.72	1.00						
CA14	0.44	0.50	0.51	0.58	0.53	0.50	0.50	0.55	0.54	0.59	0.69	0.61	0.73	1.00					
CA15	0.47	0.31	0.45	0.45	0.59	0.55	0.51	0.51	0.57	0.63	0.65	0.61	0.59	0.63	1.00				
CA16	0.46	0.41	0.54	0.52	0.57	0.60	0.53	0.52	0.60	0.65	0.57	0.62	0.54	0.62	0.68	1.00			
CA17	0.38	0.31	0.42	0.37	0.47	0.45	0.43	0.46	0.49	0.52	0.54	0.55	0.57	0.49	0.63	0.68	1.00		
CA18	0.40	0.50	0.52	0.46	0.48	0.44	0.56	0.55	0.47	0.59	0.50	0.43	0.49	0.54	0.50	0.61	0.55	1.00	
CA19	0.42	0.45	0.49	0.49	0.52	0.48	0.55	0.56	0.42	0.59	0.54	0.43	0.47	0.55	0.50	0.57	0.45	0.79	1.00
CA20	0.29	0.38	0.38	0.30	0.42	0.32	0.42	0.46	0.39	0.51	0.48	0.38	0.36	0.47	0.49	0.46	0.43	0.69	0.71
CA21	0.30	0.44	0.42	0.38	0.45	0.41	0.41	0.38	0.41	0.56	0.47	0.34	0.40	0.50	0.47	0.52	0.41	0.63	0.66
CA22	0.48	0.45	0.43	0.55	0.50	0.55	0.53	0.50	0.53	0.51	0.61	0.52	0.61	0.66	0.48	0.57	0.42	0.54	0.55
CA23	0.46	0.43	0.54	0.62	0.60	0.53	0.50	0.59	0.58	0.54	0.58	0.53	0.62	0.71	0.54	0.60	0.52	0.52	0.53
CA24	0.43	0.50	0.50	0.62	0.55	0.48	0.52	0.46	0.56	0.53	0.55	0.40	0.54	0.64	0.41	0.55	0.36	0.55	0.54
CA25	0.41	0.55	0.50	0.56	0.57	0.53	0.53	0.54	0.61	0.54	0.64	0.59	0.51	0.57	0.51	0.54	0.43	0.47	0.46
CA26	0.45	0.54	0.58	0.57	0.49	0.51	0.49	0.57	0.51	0.51	0.59	0.55	0.62	0.71	0.47	0.47	0.30	0.49	0.45
CA27	0.40	0.47	0.49	0.51	0.51	0.50	0.51	0.58	0.55	0.46	0.57	0.49	0.49	0.61	0.49	0.54	0.35	0.52	0.54
CA28	0.42	0.43	0.43	0.58	0.55	0.50	0.58	0.57	0.51	0.53	0.53	0.52	0.61	0.62	0.50	0.55	0.42	0.47	0.44
CA29	0.46	0.45	0.44	0.56	0.55	0.57	0.54	0.55	0.63	0.58	0.60	0.57	0.58	0.63	0.52	0.59	0.48	0.55	0.50
CA30	0.49	0.52	0.51	0.54	0.50	0.46	0.49	0.50	0.55	0.58	0.55	0.45	0.44	0.61	0.49	0.49	0.39	0.50	0.44
CA31	0.45	0.59	0.49	0.49	0.58	0.57	0.59	0.60	0.46	0.52	0.58	0.55	0.60	0.68	0.49	0.57	0.47	0.59	0.58
CA32	0.38	0.49	0.52	0.65	0.58	0.51	0.58	0.57	0.45	0.55	0.59	0.55	0.57	0.65	0.48	0.58	0.41	0.51	0.55
CA33	0.56	0.57	0.56	0.71	0.62	0.51	0.62	0.68	0.59	0.58	0.50	0.49	0.57	0.66	0.48	0.50	0.39	0.51	0.58
CA34	0.42	0.43	0.49	0.54	0.57	0.51	0.54	0.59	0.50	0.53	0.53	0.56	0.62	0.68	0.55	0.51	0.42	0.50	0.53
CA35	0.42	0.48	0.50	0.62	0.52	0.43	0.51	0.56	0.53	0.58	0.54	0.49	0.56	0.60	0.49	0.57	0.34	0.52	0.56
CA36	0.39	0.45	0.53	0.56	0.56	0.55	0.60	0.60	0.61	0.56	0.56	0.62	0.57	0.65	0.58	0.61	0.45	0.47	0.49
CA37	0.38	0.38	0.37	0.57	0.55	0.46	0.48	0.47	0.44	0.50	0.49	0.55	0.58	0.64	0.51	0.59	0.39	0.47	0.42

Items	CA20	CA21	CA22	CA23	CA24	CA25	CA26	CA27	CA28	CA29	CA30	CA31	CA32	CA33	CA34	CA35	CA36	CA37	
CA20	1.00																		
CA21	0.70	1.00																	
CA22	0.49	0.51	1.00																
CA23	0.46	0.51	0.73	1.00															
CA24	0.46	0.60	0.70	0.72	1.00														
CA25	0.46	0.42	0.60	0.61	0.63	1.00													
CA26	0.36	0.36	0.68	0.69	0.57	0.65	1.00												
CA27	0.54	0.55	0.66	0.65	0.64	0.65	0.64	1.00											
CA28	0.31	0.32	0.71	0.67	0.69	0.61	0.62	0.62	1.00										
CA29	0.45	0.45	0.70	0.73	0.69	0.67	0.58	0.61	0.73	1.00									
CA30	0.51	0.50	0.57	0.63	0.57	0.64	0.55	0.62	0.57	0.69	1.00								
CA31	0.48	0.55	0.59	0.61	0.59	0.58	0.49	0.59	0.58	0.66	0.70	1.00							
CA32	0.43	0.50	0.68	0.65	0.65	0.55	0.64	0.57	0.73	0.68	0.53	0.61	1.00						
CA33	0.40	0.50	0.62	0.65	0.63	0.52	0.56	0.55	0.65	0.64	0.59	0.60	0.60	1.00					
CA34	0.43	0.52	0.58	0.60	0.62	0.47	0.49	0.60	0.61	0.60	0.49	0.69	0.60	0.70	1.00				
CA35	0.45	0.52	0.68	0.7	0.72	0.54	0.62	0.62	0.71	0.65	0.59	0.58	0.76	0.66	0.65	1			
CA36	0.43	0.44	0.57	0.57	0.57	0.64	0.54	0.59	0.65	0.69	0.58	0.58	0.64	0.7	0.66	0.67	1		
CA37	0.25	0.33	0.6	0.62	0.59	0.59	0.57	0.53	0.78	0.62	0.46	0.54	0.65	0.561	0.58	0.59	0.59	1	

### Appendix 5 Principal components analysis

**Table:**

Component	Eigenvalue	Proportion	Cumulative
Comp1	19.490	0.527	0.527
Comp2	2.000	0.054	0.581
Comp3	1.741	0.047	0.628
Comp4	1.265	0.034	0.662
Comp5	0.940	0.025	0.688
Comp6	0.879	0.024	0.711
Comp7	0.782	0.021	0.732
Comp8	0.742	0.020	0.753
Comp9	0.678	0.018	0.771
Comp10	0.639	0.017	0.788
Comp11	0.629	0.017	0.805
Comp12	0.578	0.016	0.821
Comp13	0.527	0.014	0.835
Comp14	0.505	0.014	0.849
Comp15	0.454	0.012	0.861
Comp16	0.415	0.011	0.872
Comp17	0.394	0.011	0.883
Comp18	0.354	0.010	0.892
Comp19	0.352	0.010	0.902
Comp20	0.322	0.009	0.911
Comp21	0.311	0.008	0.919
Comp22	0.289	0.008	0.927
Comp23	0.280	0.008	0.934
Comp24	0.273	0.007	0.942
Comp25	0.267	0.007	0.949
Comp26	0.241	0.007	0.955
Comp27	0.230	0.006	0.962
Comp28	0.199	0.005	0.967
Comp29	0.182	0.005	0.972
Comp30	0.176	0.005	0.977
Comp31	0.171	0.005	0.981
Comp32	0.148	0.004	0.985
Comp33	0.136	0.004	0.989
Comp34	0.125	0.003	0.992
Comp35	0.110	0.003	0.995
Comp36	0.090	0.002	0.998
Comp37	0.083	0.002	1.000

### Appendix 7 Parallel analysis graph

Figure illustrates the expected eigenvalues (the solid line) and parallel analysis (the dotted line), PCA = Principal Components Analysis.

### Appendix 8 Parallel analysis table

**Table:**

Component	PCA	PA	Difference
1	19.490	2.065	17.425
2	2.000	1.920	0.080
3	1.741	1.819	− 0.078
4	1.265	1.732	− 0.467
5	0.940	1.654	− 0.713
6	0.879	1.584	− 0.705
7	0.782	1.517	− 0.734
8	0.742	1.454	− 0.712
9	0.678	1.394	− 0.716
10	0.639	1.340	− 0.701
11	0.629	1.287	− 0.658
12	0.578	1.236	− 0.658
13	0.527	1.187	− 0.660
14	0.505	1.141	− 0.636
15	0.454	1.095	− 0.641
16	0.415	1.051	− 0.636
17	0.394	1.007	− 0.613
18	0.354	0.967	− 0.613
19	0.352	0.926	− 0.574
20	0.322	0.887	− 0.564
21	0.311	0.849	− 0.538
22	0.289	0.812	− 0.523
23	0.280	0.776	− 0.496
24	0.273	0.740	− 0.467
25	0.267	0.704	− 0.438
26	0.241	0.670	− 0.429
27	0.230	0.636	− 0.406
28	0.199	0.603	− 0.404
29	0.182	0.570	− 0.388
30	0.176	0.538	− 0.362
31	0.171	0.506	− 0.335
32	0.148	0.474	− 0.325
33	0.136	0.442	− 0.305
34	0.125	0.408	− 0.283
35	0.110	0.375	− 0.265
36	0.090	0.338	− 0.248
37	0.083	0.296	− 0.212
